# Management of thrombocythemia

**DOI:** 10.12688/f1000research.5361.1

**Published:** 2014-09-29

**Authors:** Krisstina Gowin, Ruben Mesa

**Affiliations:** 1 Department of Hematology and Medical Oncology, Mayo Clinic Arizona, Scottsdale, AZ, 85259, USA; 2 Division of Hematology and Medical Oncology, Mayo Clinic Cancer Center, Scottsdale, AZ, 85259, USA

## Abstract

Essential thrombocythemia is a clonal myeloproliferative neoplasm characterized by an elevated platelet count, the potential for both microvascular and macrovascular sequelae, and a risk for transformation to myelofibrosis or acute myeloid leukemia. A systematic and detailed initial analysis is essential for accurate diagnosis of essential thrombocythemia, as many etiologies are reactive and benign. Once a diagnosis has been made, risk stratification and symptom assessment are vital to guide the subsequent therapy. Treatment may be required in high-risk disease, such as in cases of advanced age or prior thrombotic events. Systemic therapy is aimed at reducing the thrombotic risk and includes daily low dose aspirin and in some patients, cytoreductive therapy.  Currently, the first line cytoreductive therapy includes hydroxyurea or pegylated interferon, with a phase III clinical trial underway comparing these two important agents. Anagrelide and clinical trials are reserved for refractory or intolerant patients. Looking to the future, new therapies including Janus kinase 2 (JAK2) and telomerase inhibitors are promising and may become valuable to the treatment armamentarium for those afflicted with essential thrombocythemia.

## Introduction

Thrombocythemia, or elevation in platelet count (i.e. greater than 450 × 10 (9)/L), is a common observation for internists and hematologists alike. Causes may be secondary or “acquired” in contrast to primary thrombocythemia, meaning that the pathogenesis lies within the abnormal marrow itself. Essential thrombocythemia (ET), one of the myeloproliferative neoplasms (MPNs), is an aberration within the bone marrow and its microenvironment leading to clonal proliferation of the megakaryocytic lineage within the marrow and, ultimately, to peripheral blood thrombocythemia. Unlike secondary thrombocythemia, ET is associated with thrombotic and hemorrhagic complications and requires systemic medical therapy in high-risk patients. In this brief article, we discuss the diagnostic strategy of thrombocytosis with particular attention paid to essential thrombocythemia. Subsequently, the clinical manifestations of ET are examined and the assessment of disease burden is reviewed. The history of therapeutics for ET is reviewed with consideration to the current rationale for therapeutic decision-making.

## Uncovering the etiology

Elucidating the etiology of thrombocythemia is of utmost importance prior to any therapeutic decision-making. Certainly, clonal bone marrow diseases such MPNs should be considered. However, such a diagnosis can only be considered after eliminating secondary contributions to the elevated platelet count. Many chronic and acute processes cause stimulation and up-regulation of bone marrow stem cells including infection, malignancy, iron deficiency, prior splenectomy, and recent trauma or surgery. (see Table 1). A careful examination of iron status, inflammatory markers, and age appropriate malignancy screen is imperative. History and physical exam, such as a history of gastrointestinal bleeding, rheumatologic disease or the presence of splenomegaly on exam can lend clues as to underlying etiologies. Once secondary causes are excluded, evaluation for an underlying clonal myeloproliferative disorder can commence.

**Table 1.** Causes of thrombocythemia.

**Secondary** **thrombocythemia**	– Infection – Post surgical status, particularly orthopedic procedures – Malignancy – Post Splenectomy – Iron deficiency – Hemolytic anemia – B12 deficiency – Immune thrombocytopenia purpura (ITP) rebound effect – Severe burns – Rheumatologic disorders: rheumatoid arthritis (RA), systemic lupus erythematosus (SLE), celiac sprue – Medications
**Primary** **thrombocythemia**	– Philadelphia negative myeloproliferative neoplasms (PV, ET, MF) – Chronic Myeloid Leukemia (CML) – Chronic Myelomonocytic Leukemia (CMML) – Myelodysplastic syndrome (MDS) and MPN overlap syndrome

## Establishing a diagnosis

MPNs such as ET, polycythemia vera (PV), and myelofibrosis (MF) are Philadelphia negative clonal disorders of the bone marrow ^1^. When attempting to establish a diagnosis of MPN, mutational status can be quite helpful ^2^. In 2005, a landmark discovery identified a gain of function mutation, *JAKV617F*, as being an essential mutational driver in many MPNs ^3– 5^. In ET, approximately 50% of patients will harbor the *JAKV617F* mutation. PCR based assays for the JAK2 mutation, from either peripheral blood or marrow, are commercially available. Since the *JAKV617F* discovery, other molecular breakthroughs have contributed not only to our knowledge of pathogenesis in MPNs but also how we diagnose them. In the majority of *JAKV617F* wild type patients, the *CALR* (calreticulin gene *)* mutation ^6^ may be detected and now is a widely available assay. Additionally, *MPL* (myeloproliferative leukemia gene) mutations are detected in a small percentage (<5%) of those afflicted with ET ^7, 8^. Although mutation analysis is critical for the evaluation of a suspected MPN, it is not sufficient for diagnosis. A bone marrow biopsy must be obtained and possess features consistent with ET, such as megakaryocytic hyperplasia. Additionally, assessment of cytogenetics, baseline karyotype, reticulin fibrosis, and blast percentage should be performed. Mutational status for other myeloid diseases must be evaluated and negative including the *BCR-ABL*, i.e. “the Philadelphia chromosome”, and fluorescence in situ hybridization (FISH) for myelodysplastic syndrome (MDS) panel to exclude the diagnosis of CML and MDS, respectively (see Table 2) ^2^.

**Table 2.** WHO 2008 criteria for essential thrombocythemia (ET) ^2^.

Diagnosis requires meeting ALL 4 criteria:
1. Sustained platelet count ≥450 × 10 (9)/L 2. Bone marrow biopsy showing proliferation mainly of megakaryocytic lineage with increased numbers of enlarged, mature megakaryocytes. No significant increase or left-shift of neutrophil granulopoiesis or erythropoiesis. 3. No meeting WHO criteria for polycythemia vera, primary myelofibrosis, *BCR-ABL* positive CML, MDS, or other myeloid neoplasm. 4. Demonstration of *JAK2 V617F* or other clonal marker, or in the absence of *JAK2 V617F*, no evidence of reactive thrombocytosis.

## Assessing symptom burden

The presentation of MPN may be quite variable. A large proportion of those afflicted with ET are completely asymptomatic at presentation. Unfortunately, approximately 50% of patients with ET do possess some form of systemic manifestation of the disease and experience a substantial impact on their quality of life ^9^. Common symptoms may include those from microvascular complications such as headache, dizziness, paresthesia, livedo reticularis, erythromelalgia, and visual changes. Others may present with the more dreaded macrovascular complications such as myocardial infarction, stroke, or pulmonary embolus. Additionally, constitutional symptoms may be prevalent with symptoms of fatigue, night sweats, and weight loss; particularly in those transitioning to a more myelofibrotic state. In 2007, a group of researchers set out to create and validate a symptom assessment tool specific to the MPN population ^10^. Due to this effort, the MPN-Symptom Assessment Form (SAF) ^11^ is now available and validated for use in this patient population and has proven to be an invaluable tool in the assessment and management of ET. Though use of the MPN-SAF a subset of ET patients were identified who possess a significant symptomatic burden, with fatigue and microvascular complications being the most prevalent ^10^. For the treating clinician, the MPN-SAF can be utilized to assess baseline symptomatology and help guide initial therapeutic decision-making as well as gauge subsequent response to therapy.

## Risk assessment

Deciding when to initiate therapy in ET may be complex and represents a unique challenge in the treatment of MPNs. A thrombotic risk assessment is necessary to evaluate whether initiation of cytoreduction is warranted ^12, 13^. The presence of high-risk features ^14^, such as age greater than 60 years and a prior history of thrombosis, is predictive of future complications and generally prompts the clinician to employ cytoreduction. Additionally, concurrent cardiovascular risk factors ^15^, *JAK V617F* mutational status and allelic burden ^16, 17^, and the presence of leukocytosis ^18^ may increase the thrombotic risk potential and contribute to a clinician’s decision to initiate therapy. The presence of a heavy symptom burden may also provide more impetus to employ cytoreduction in afflicted patients who are otherwise in a low risk category. An international prognostic model for ET was developed in 2012 by Passamonti *et al.* and is helpful to ascertain risk and give valuable prognostic information to the treating physician (see Table 3) ^13^. The treatment goal is improvement in disease related symptoms in addition to normalization of the platelet count to decrease thrombotic risk potential. Typically, the minimal effective dose is utilized to limit treatment-associated toxicity. In those with low-risk asymptomatic disease, simple observation is appropriate.

**Table 3.** Essential thrombocythemia risk assessment per IPSET ^13^.

**Prognostic features in ET**	– Age >60 years (2 points) – Prior history of thrombosis (1 point) – Leukocytes >11 × 10 (9)/L (1 point) **Risk group:** **0:** Low **1-2:** Intermediate **3-4:** High

## Initial systemic therapy

In 2004, a European group investigated the use of aspirin for the prevention of thrombotic complications in PV and found that daily low dose aspirin can safely prevent thrombotic complications in those who have no contraindications to such treatment ^19^. Since this landmark study, it is standard practice to administer daily low dose aspirin to all those with high-risk ET. In those with very high initial platelet counts, greater than 1,500/microL, an acquired Von Willebrand deficit may occur and increase risk for hemorrhagic complications. Because of this, some practitioners may elect to cytoreduce prior to aspirin initiation. Currently, first line cytoreductive therapy is a choice amongst three agents: hydroxyurea, anagrelide, and pegylated interferon. Fortunately, recent trials have clarified some therapeutic nuances of each choice. Hydroxyurea is a traditional treatment for preventing thrombosis in ET since Cortelazzo published on its efficacy in 1995 ^20^. Later, anagrelide was approved for control of thrombocytosis based on single arm studies ^21^. Subsequently, a conundrum was raised as to which agent was superior and preferential in first line therapy. In 2005, Harrison *et al.* sought to answer this with a randomized comparison of hydroxyurea to anagrelide ^22^. In this study, hydroxyurea was found to be superior to anagrelide in terms of rate of arterial thrombosis, serious hemorrhage, and transformation to myelofibrosis, but was inferior in terms of rates of venous thrombosis. Consequently, hydroxyurea became standard first line therapy, with anagrelide being reserved for second line treatment. In 2008, pegylated interferon, a more tolerable form of interferon, was demonstrated to induce hematologic and molecular responses in ET ^23, 24^. As an added benefit, pegylated interferon has been shown to retard progression towards fibrosis in some studies ^25, 26^ however this remains controversial and is an area of ongoing investigation. Currently, it is still unknown whether hydroxyurea or pegylated interferon represents the best initial treatment strategy. The Myeloproliferative Disorders Research Consortium (MPD-RC) is conducting a phase III international study to evaluate the efficacy, safety, and tolerability of hydroxyurea versus pegylated interferon in frontline therapy for ET/PV. (clinicaltrials.gov: NCTO1259817). Additionally, it is important to mention that interferon therapy is safe in pregnancy, unlike hydroxyurea and anagrelide and thus, pegylated interferon is the preferred agent in this patient population or those who wish to become pregnant.

## Second line therapy

In those who are intolerant or resistant to initial therapy a therapeutic switch is indicated and is largely guided by first line choices. A common practice is to progress through the first line cytoreductive agents, with no data directing the sequence of therapies. Aspirin is continued throughout if not contraindicated. The duration of therapy is typically lifelong, with the goal of treatment being hemorrhagic and thrombotic risk reduction, as well as retardation of disease progression. For those who are intolerant to or progressed on all approved agents, clinical trials should be considered. Novel therapeutics, particularly JAK inhibitors, offer a valuable addition to the treatment armamentarium and are available via clinical trial for ET. Moreover, other drug classes such as telomerase inhibitors are promising for the future treatment of ET. Often, in those with very proliferative disease (i.e. platelet count >2000 × 10(9)/L), an effective combination therapeutic approach is used. Hydroxyurea and anagrelide, for example, can be used concurrently for optimal cytoreduction and greater tolerability, as the dosage of each is lower in combination than with single agent therapy alone.

## Monitoring for progression

A minority of patients progress to myelofibrosis or acute myeloid leukemia (AML) ^27^. Practitioners should pay careful attention to the patient’s symptom burden, peripheral blood counts, and cytogenetic analysis for clues indicating progression. The development of increased constitutional symptoms such as progressive splenomegaly, fever, weight loss, early satiety, and bone pain in conjunction with a trend towards either new cytopenia or increased rate of proliferative disease increases clinical suspicion of a post ET-myelofibrosis. Conversely, those with new blasts on peripheral smear and/or marrow and new cytogenetic complexity should be evaluated for MPN blast phase or AML ^28^.

## Conclusion

In evaluating cases of thrombocythemia, it is essential to exclude both reactive processes and other chronic myeloid disorders prior to making the diagnosis of essential thrombocythemia. Mutational analysis is helpful in making the diagnosis and the well-informed clinician can consider *JAKV617F* and if wild type subsequent *MPL,* and *CALR* assessment in new patient evaluations. In ET patients with high-risk disease, aspirin plus either hydroxyurea versus pegylated interferon is the standard first line therapy. Anagrelide is appropriate as an adjunct to therapy or for second line usage. Clinical trial enrollment is imperative to answer outstanding questions regarding safety, tolerability, and efficacy of alternative therapies ^29^ including JAK2 and telomerase inhibitors, both of which have demonstrated promising early results in the treatment of ET.
