# Supplementary material for: Management of thrombocythemia
Source: F1000Res. 2014 Sep 29;3:227. [Version 1] doi: 10.12688/f1000research.5361.1 (PMC4197738; doi:10.12688/f1000research.5361.1)
Supplement: Peer review history

# Peer review: Referee response for version 1

**DOI:** 10.5256/f1000research.5723.r6243

**Published:** 2014-10-13

**Authors:** Ann Mullally

**Affiliations:** 1 Division of Hematology, Department of Medicine, Brigham and Women's Hospital, Harvard Medical School, Boston, USA

Nice summary of clinical management of ET.

I have read this submission. I believe that I have an appropriate level of expertise to confirm that it is of an acceptable scientific standard.

# Peer review: Referee response for version 1

**DOI:** 10.5256/f1000research.5723.r6242

**Published:** 2014-10-03

**Authors:** Laura C. Michaelis

**Affiliations:** 1 Hematologic Malignancies Program, Division of Hematology/Oncology, Medical College of Wisconsin, Froedtert Hospital, Milwaukee, USA

This is a well-written review that addresses some of the key considerations in the diagnosis and treatment of ET. The authors are right to emphasize the importance of a bone marrow biopsy in diagnosis, as it is important to exclude early PMF, which can have a different disease trajectory. In addition, practitioners should also be aware of the disproportionately high rates of splanchnic vein or mesenteric thromboses, which can be a presenting symptom in these patients and should prompt clinicians to search for MPNs.

I have read this submission. I believe that I have an appropriate level of expertise to confirm that it is of an acceptable scientific standard.
